# Elemene injection combined with transcatheter arterial chemoembolization for unresectable hepatocellular carcinoma: A meta-analysis

**DOI:** 10.1097/MD.0000000000017813

**Published:** 2019-11-01

**Authors:** Yuan Yao, Jianjian Chen, Dechao Jiao, Yahua Li, Xueliang Zhou, Xinwei Han

**Affiliations:** Department of Interventional Radiology, the First Affiliated Hospital of Zhengzhou University, Zhengzhou, PR China.

**Keywords:** elemene, hepatocellular carcinoma, TACE, traditional Chinese medicine

## Abstract

**Background:**

To evaluate the short-term efficacy, long-term efficacy, and adverse events (AEs) of elemene plus transcatheter arterial chemoembolization (TACE) in comparison with TACE alone for the treatment of hepatocellular carcinoma (HCC).

**Methods:**

PubMed, EMBASE, the Cochrane Library, the Chinese Scientific Journal Full-text Database, Wanfang Data, CBM, and VIP were searched by 2 reviewers using the same search strategy for clinical studies on elemene plus TACE in the treatment of HCC. These articles were screened according to pre-established inclusion and exclusion criteria, and the qualities of the included studies were assessed using the Newcastle–Ottawa scale. The primary outcomes were the objective response rate (ORR), the 1-year survival rate and AEs. Review Manager 5.3 and Stata 15.0 were used for the meta-analysis.

**Results:**

A total of 10 studies involving 543 patients (TACE + elemene = 277, TACE alone = 266) were included. The results showed that the ORR was significantly improved in the combined treatment group compared to the TACE alone group (odds ratio [OR] = 2.72, 95% confidence interval [CI]: 1.84–4.00, *P* < .05). TACE + elemene significantly increased the 1-year survival rate (OR = 2.79, 95% CI: 1.58–4.95, *P* < .05). We also found no significant difference in gastrointestinal reactions (OR = 0.97, 95% CI: 0.57–1.64, *P* = .90), fever (OR = 0.80, 95% CI: 0.37–1.71, *P* = .56), or bone marrow suppression (OR = 0.73, 95% CI: 0.44–1.22, *P* = .23) between the 2 groups.

**Conclusion:**

Based on current findings, TACE + elemene injection may improve the ORR and the 1-year survival rate for HCC patients compared to TACE alone. Arterial perfusion may be superior to intravenous guttae.

## 1 Introduction

According to recent statistics, liver cancer is the second most frequently diagnosed cancer among men and the leading cause of cancer-related deaths.^[1]^ Hepatocellular carcinoma (HCC) is the most common type of liver cancer, and liver cirrhosis is a major factor in the development of HCC that may affect the surgical approach and the pharmacokinetics of chemotherapy drugs.^[2]^ Hepatic resection and liver transplantation are possible curative treatments for early-stage HCC patients, while transcatheter arterial chemoembolization (TACE) is an established local therapy for unresectable HCC or a preoperative treatment.^[3,4]^ Moreover, more than 70% of patients are diagnosed in advanced stages.^[5]^ TACE is an effective and alternative treatment for intermediate or advanced HCC patients.^[6]^

The principle of TACE is the intra-arterial injection of a chemotherapeutic drug such as platinum or anthracyclines followed by embolization of the blood vessel, which will result in a strong cytotoxic effect enhanced by ischemia.^[7]^ Many researchers have proven that TACE combined with other treatments, such as molecular targeted drugs, immunotherapy drugs, radioactive particles, and traditional Chinese medicine, may have better clinical effects compared to those from TACE alone.^[8–10]^

Elemene is the active ingredient extracted from the traditional Chinese medicine Curcuma Wenyujin. The main ingredient is β-elemene, which has proven to be effective in various primary or secondary malignant pleural cavities, peritoneal effusion, and liver, lung, digestive tract, and brain tumors in clinical trials. β-Elemene inhibits cell proliferation, prevents the cell cycle, induces apoptosis, exerts antiangiogenic and antimetastatic effects, reverses multidrug resistance, and enhances the immune system to exert its effects.^[11]^ Elemene combined with TACE has good potential in the theoretical treatment of HCC. However, combination therapy is still controversial, lacking related objective evidence, and the local effect of TACE plus elemene remains uncertain. The purpose of this meta-analysis was to compare the efficacy and adverse reactions of elemene injection combined with TACE and TACE alone to provide evidence for clinical decision-making.

## 2 Materials and methods

### 2.1 Literature sources and search strategy

PubMed, EMBASE, the Cochrane Library, the Chinese Scientific Journal Full-text Database, Wanfang Data, CBM, and VIP were searched by 2 reviewers using the same search strategy for clinical studies on elemene combined with TACE in the treatment of HCC. The following terms were included in the search strategy: “hepatocellular carcinoma,” “hepatocellular cancer” AND “Elemene” AND “transcatheter arterial chemoembolization,” “TACE.” The search included articles published until April 10th, 2019, with no lower date limit. No language restrictions were applied to this search.

### 2.2 Inclusion criteria

The inclusion criteria were as follows:

(1) Study subjects: all enrolled patients had a clear pathological diagnosis and clear TACE indications;
(2) Study group: the experimental group was treated with TACE combined with elemene injection, and the control group underwent TACE alone; and
(3) The primary outcomes were short-term efficacy (according to the short-term efficacy criteria of Response Evaluation Criteria in Solid Tumors [RECIST] or World Health Organization [WHO] solid tumors, objective response rate [ORR] = complete response [CR] + partial response [PR]) and adverse events (according to the WHO common adverse reactions grading criteria).

### 2.3 Exclusion criteria

The exclusion criteria were as follows:

(1) The enrolled patients did not explicitly mention the clinicopathological diagnosis or TACE indications;
(2) None of the study indicators mentioned short-term efficacy or evaluation criteria;
(3) Incorrect or incomplete data; and
(4) Recurrent literature.

### 2.4 Data extraction

Data were extracted by 2 authors using a standardized form and checked independently by 2 reviewers (Yao Y and Chen JJ). If necessary, a third author (Zhou XL) participated in the resolution differences. The following content was extracted:

(1) Information about the publication of the included literature, such as the publication year, the first author and the source of the subjects;
(2) The specific course of medication and the number of cases in the experimental and control groups; and
(3) Study outcomes included the ORR, adverse events (gastrointestinal reactions and bone marrow suppression, etc), and the 1-year survival rate.

### 2.5 Quality assessments

All included articles were nonrandomized studies, and the Newcastle–Ottawa scale (NOS) was adopted as the standard to evaluate the quality of the included studies. The quality of the included studies was assessed according to the following criteria:

(1) Is the case definition adequate?;
(2) Representativeness of the cases;
(3) Selection of controls;
(4) Definition of controls;
(5) Selection of the most important factor;
(6) Any additional factor;
(7) Ascertainment of exposure;
(8) Same method of ascertainment for cases and controls; and
(9) The nonresponse rate.

Each item was assessed and scored with stars. The final star is presented in the quality assessment table (studies with scores >7 were considered as having a low risk of bias, scores of 4–6 were considered as having a moderate risk of bias, and scores <4 were considered as having a high risk of bias).

### 2.6 Statistical analysis

RevMan (version 5.3) and Stata 15.0 were used for data collation and analysis, and the OR value and 95% confidence interval (95% CI) of each study were calculated. By using RevMan software, we combined and summarized the results of various studies in the form of forest plot, including subgroup analysis, etc. Meanwhile, Stata 15.0 software was used to draw funnel plot and sensitivity analysis graph, and the funnel plot adopted Begg test statistical method and calculated statistical quantity. Heterogeneity was assessed by the Chi-square test at a level of *α* = 0.1, and the *I*^2^-index was used for the quantitative analysis of heterogeneity. A *P*-value < .05 and an *I*^2^ > 50% were considered to indicate significant heterogeneity. The results of the study without significant heterogeneity were analyzed in combination with the fixed-effects model; otherwise, the random-effects model *Z* test was selected after explaining the possible causes of heterogeneity, and the difference was statistically significant at *P* < .05.

## 3 Results

### 3.1 General characteristics and quality evaluation

**Figure 1:**
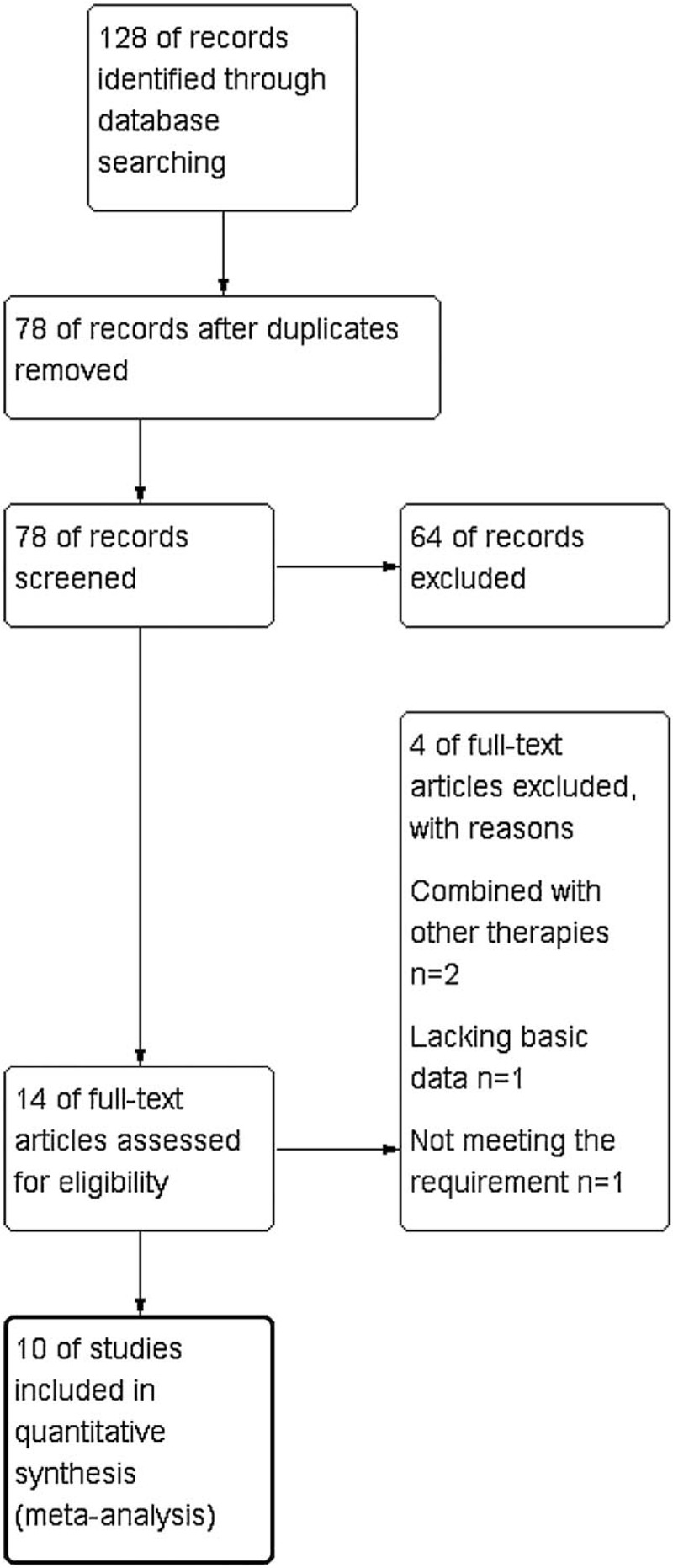
Flow diagram of the process for the identification of eligible studies.

The search strategy identified 128 relevant studies, of which 50 were duplicates. A total of 64 references were excluded after the titles and abstracts were screened; then, 4 studies were excluded for other reasons. Finally, 10 studies^[12–21]^ were included in the meta-analysis. The flow diagram of the process is shown in Figure 1. Table 1 presents the basic characteristics of the included studies. A total of 543 patients were included in this study, of whom 277 were treated with TACE + elemene, and 266 were treated with TACE alone. The quality of case-control studies was assessed by the NOS, which included the evaluation of risk of bias in the selection of study groups, comparability of groups, and ascertainment of the exposure or outcome of interest. Specific details on the risk of bias in the included studies are reported in Table 2. Ten articles were published in Chinese journals, and these journals have high credibility in the HCC field in China. The scores ranged from 6 to 8, indicating that these articles were of high quality.

**Table 1:**
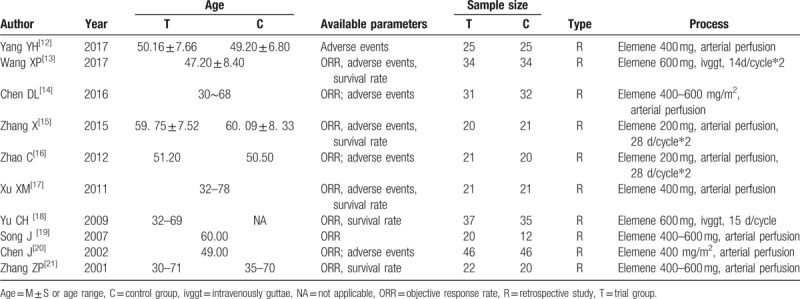
Basic characteristics of included studies.

**Table 2:**
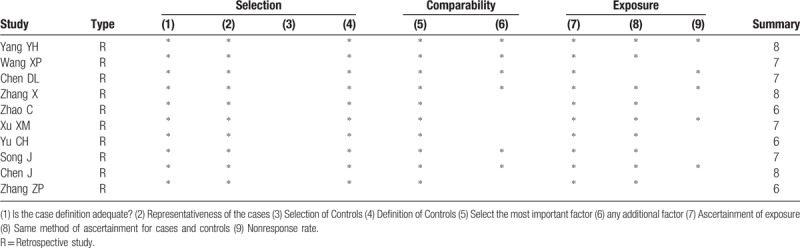
Quality assessment of the included studies.

### 3.2 Objective response rate

**Figure 2:**
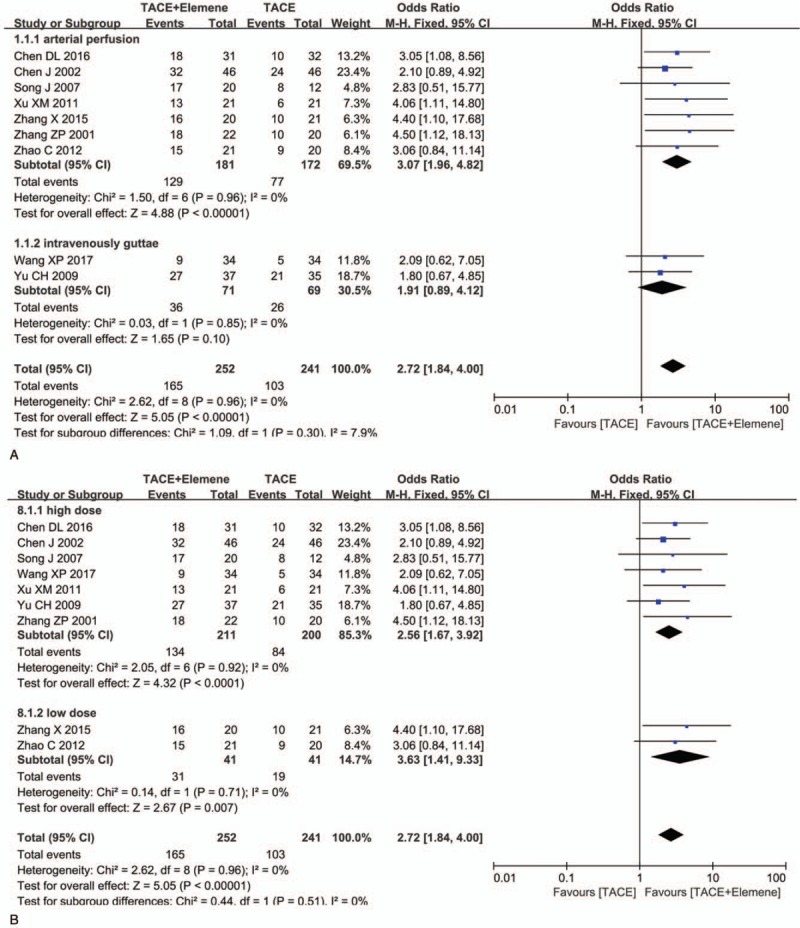
The forest plot of odds ratio for evaluation of objective response rate. (A) Subgroup analysis result of ORR according to the delivery way; (B) subgroup analysis result of ORR according to the single dose. CI = confidence interval, OR = odds ratio, ORR = objective response rate.

Nine of the included 10 studies^[13–21]^ reported the ORR. The objective response to treatment was classified as a CR, a PR, stable disease, and progressive disease, and the ORR was defined as PR + CR. The results showed that the ORR was significantly improved in the TACE + elemene compared to the TACE alone group (odds ratio [OR] = 2.72, 95% CI: 1.84–4.00, *P* < .05). No heterogeneity was detected among these trials (*P* = .96, *I*^2^ = 0). A subgroup analysis was performed according to the delivery method (the arterial perfusion subgroup and the intravenous drip subgroup). The results of the arterial perfusion subgroup analysis revealed a higher ORR in the combined group (OR = 3.07, 95% CI: 1.96–4.82, *P* < .05), while the ORR in the intravenous guttae subgroup did not have statistical significance between these 2 groups (OR = 1.91, 95% CI: 0.89–4.12, *P* = .1). According to the single dose, patients were divided into 2 subgroups (low dose: single dose 200 mL, 2 courses of treatment; high dose: single dose beyond 400 mL, 1 course of treatment.) Both subgroups showed statistically significant differences (low dose subgroup: OR = 3.63, 95% CI: 1.41–9.33, *P* < .05, *I*^2^ = 0; high dose subgroup: OR = 2.56, 95% CI: 1.67–3.92, *P* < .05, *I*^2^ = 0) (Fig. 2).

### 3.3 One-year survival rate

**Figure 3:**
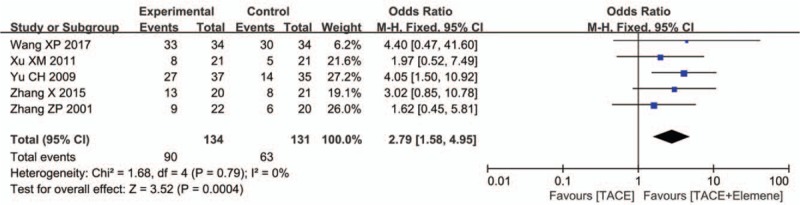
The forest plot of odds ratio for evaluation of 1-yr survival rate.

Five of 8 retrospective studies^[13,15,17,18,21]^ reported the 1-year survival rate on 265 patients. No heterogeneity was found among these 5 studies (*P* = .79, *I*^2^ = 0). A fixed-effects model was adopted for the meta-analysis. We found that TACE + elemene significantly improved the 1-year survival rate compared to TACE alone (OR = 2.79, 95% CI: 1.58–4.95, *P* < .05) (Fig. 3).

### 3.4 Adverse events

**Figure 4:**
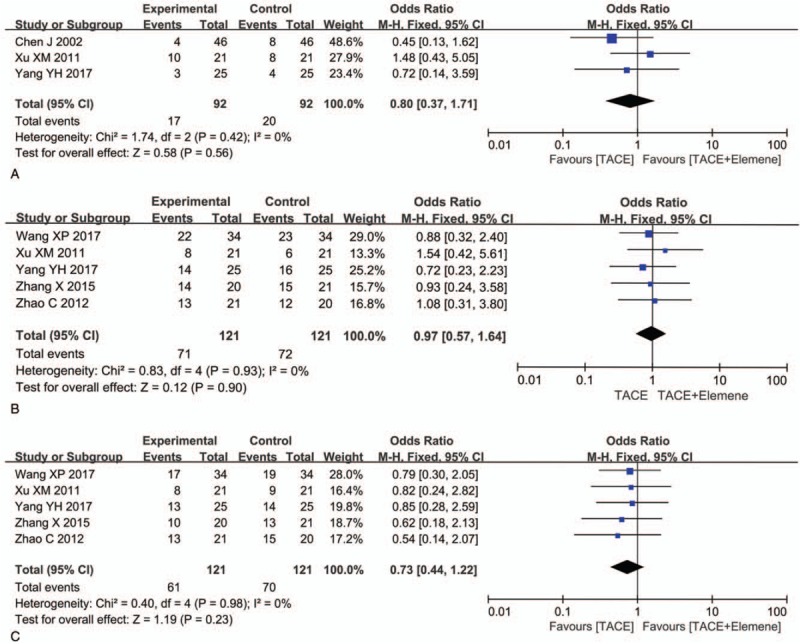
The forest plot of odds ratio for evaluation of adverse events. (A) fever, (B) gastrointestinal reactions, and (C) bone marrow suppression.

In the TACE + elemene group, no serious adverse events or deaths were reported related to Chinese medicine. The common adverse events caused by TACE included bone marrow suppression, gastrointestinal reactions (abdominal pain, nausea, and vomiting), fever, and liver dysfunction (transaminase elevations), but these events were mild, and all the adverse events were relieved after symptomatic treatment. Five articles^[12,13,15–17]^ reported specific data on bone marrow suppression (OR = 0.73, 95% CI: 0.44–1.22, *P* = .23), 5 articles^[12,13,15–17]^ reported gastrointestinal reactions (OR = 0.97, 95% CI: 0.57–1.64, *P* = .90), and 3 studies^[12,17,20]^ showed valid data on fever (OR = 0.80, 95% CI: 0.37–1.71, *P* = .56). In terms of adverse events, there was no statistically significant difference between the experimental group and the control group. TACE + elemene may not increase the probability of adverse events. To a certain extent, there is a tendency to alleviate bone marrow suppression (Fig. 4).

### 3.5 Publication bias

**Figure 5:**
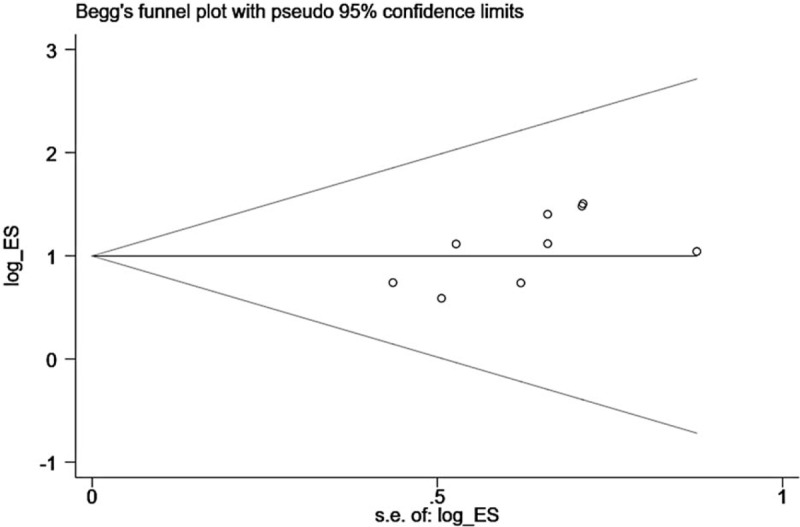
Funnel plot for objective response rate.

We drew a funnel plot by using Stata 15.0 software (Fig. 5). Begg test was used to evaluate publication bias for the ORR, and the result suggested that there was no obvious publication bias (*P* = .076). Publication bias was not evaluated for the 1-year survival rate or adverse events because the number of included articles concerning these outcomes was too few; therefore, the Begg test was inappropriate.^[22]^ The evaluation results were not very stable due to the insufficiency of the included studies, and more studies are needed to modify and supplement the results.

### 3.6 Sensitivity analysis

**Figure 6:**
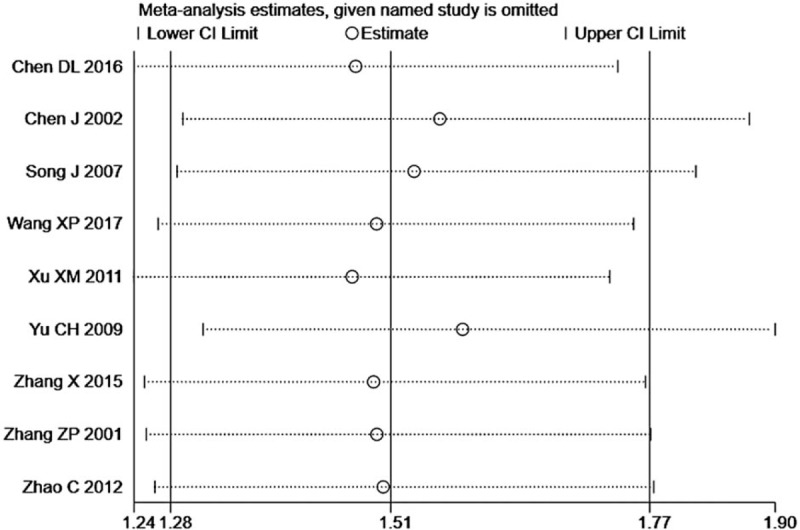
ORR sensitivity analysis diagram. ORR = objective response rate.

Regarding deleting a certain study, the point estimation of the combined value fell within the range of 2 effective lines and was close to the total combined value, which suggested no significant heterogeneity (Fig. 6). Furthermore, the results of the forest plot (*I*^2^ = 0*, P* = .96) also showed that no heterogeneity or deviation existed among the included studies.

## 4 Discussion

Ten studies were included in the meta-analysis, and a total of 543 patients were included, of whom 277 were treated with TACE + elemene, and 266 were treated with TACE alone. From the combined results, the ORR and the 1-year survival rate of TACE combined with elemene were significantly higher than those of TACE alone, while there was no significant difference in adverse reactions between these 2 groups. The results of the meta-analysis showed that compared with TACE alone, the ORR (OR = 2.72, *P* < .05; arterial perfusion subgroup: OR = 3.07, *P* < .05; intravenously guttae subgroup: OR = 1.91, *P* > .05) and the 1-year survival rate (OR = 2.79, *P* < .05) were significantly improved following treatment with elemene plus TACE. Furthermore, elemene did not increase the incidence of gastrointestinal reactions (OR = 0.97, *P* = .90) fever (OR = 0.80, *P* = .56), or bone marrow suppression (OR = 0.73, *P* = .23). In contrast, the combined group showed a trend of reduced bone marrow suppression, which may be related to elemene's role in enhancing the immune function of the body. According to the subgroup analysis (Fig. 2A), arterial infusion is significantly better than intravenous infusion, and the ORR may not be improved by intravenous infusion compared with that from TACE alone (*P* > .05). It should be noted that different delivery routes may directly affect the therapeutic effect. Clinicians should try to use arterial perfusion instead of intravenous guttae. On the other hand, the subgroup analysis of single does showed that it may be better to give a low dose in 2 courses than a large dose in 1 course, but there is no difference in the total dose (Fig. 2B). More clinical trials are needed to verify these results.

Although several researchers have reported that TACE is effective in the treatment of HCC and the clinical effect of TACE on improving the ORR and survival rates for advanced HCC patients, few have evaluated the effect and safety of TACE + elemene. This was the first meta-analysis to identify nearly all studies about TACE plus elemene for the treatment for HCC, evaluate the clinical effectiveness and provide a foundation for combined therapy in the future. However, it is undeniable whether there will be new adverse reactions in this treatment mode and/or whether it will increase the financial burden of patients, as the included literature has not reported on these issues. In view of the current treatment status, many researchers have also confirmed that TACE combined therapy has significantly improved the local control rate, survival rate, and quality of life of patients. The combined therapies are complementary and have improved the development of TACE alone.^[23–26]^ On the other hand, the controversial aspect is the mechanism of action of elemene, which is ubiquitous in traditional Chinese medicine. Several researchers have tried to unveil the mystery of elemene by studying it at the cellular and molecular levels.

Elemene is an antitumor drug independently developed in China, and β-elemene is the main component of its clinical preparation, with strong anticancer properties and low toxicity.^[27]^ In previous studies, elemene has played an important role in the treatment of a variety of tumors, including lung cancer,^[28]^ malignant pleural effusion,^[29]^ and Burkitt's lymphoma.^[30]^ Wu et al^[31]^ reported that elemene induced cell apoptosis, inhibited the cell cycle, and reversed GSTP1 gene methylation in QGY7703 cells. Mao et al^[32]^ reported that β-elemene injection could inhibit the proliferation of hepatoma HepG2 cells and induce cell apoptosis, the mechanism of which might be partly related to the downregulation of alpha-tubulin and the inhibition of microtubular polymerization. All these findings are worth exploring further.

This meta-analysis has the following strengths:

(1) This is the first meta-analysis in nearly 2 decades that combined TACE + elemene therapy and included as many studies as possible;
(2) This meta-analysis provides an interpretation of the controversial aspects (effectiveness of combination therapy) and may provide a reference for new clinical treatments (delivery routes);
(3) The risk of publication bias was low among the included studies; and
(4) No significant heterogeneity or deviation existed by performing the sensitivity analysis.

However, this study has the following limitations:

(1) A lack of high-quality randomized controlled trials (RCTs) (RCTs are the highest quality studies for a meta-analysis);
(2) The mechanism of elemene action is not very clear and needs further basic research;
(3) Due to the limited follow-up time, there may be other complications (a longer follow-up is needed to confirm the efficacy of the combined treatment);
(4) A lack of uniform standards for delivery routes and doses, which may lead to a bias of results; and
(5) All the studies included came from China, and there may be regional bias.

## 5 Conclusion

In conclusion, this meta-analysis suggests that TACE + elemene is superior to TACE alone. Arterial perfusion may be superior to intravenous guttae. Further studies are needed to make more comprehensive comparisons in the future.

## Author contributions

**Conceptualization:** Yuan Yao.

**Data curation:** Yahua Li.

**Formal analysis:** Jianjian Chen, Yahua Li.

**Investigation:** Jianjian Chen, Xueliang Zhou.

**Methodology:** Yuan Yao, Jianjian Chen, Yahua Li.

**Software:** Dechao Jiao, Xueliang Zhou.

**Supervision:** Dechao Jiao, Xinwei Han.

**Writing – original draft:** Yuan Yao.
